# Overstretching Expectations May Endanger the Success of the “Millennium Surgery”

**DOI:** 10.3389/fbioe.2022.789629

**Published:** 2022-02-14

**Authors:** Alwina Bender, Philipp Damm, Hagen Hommel, Georg N. Duda

**Affiliations:** ^1^ Berlin Institute of Health at Charité-Universitätsmedizin Berlin, Julius-Wolff-Institute, Berlin, Germany; ^2^ Department of Orthopaedics, Märkisch-Oderland Hospital, Brandenburg Medical School Theodor Fontane, Wriezen, Germany

**Keywords:** total hip arthroplasty, loads, sport activities, activity of daily living, age

## Abstract

Total hip arthroplasty (THA) is an extremely successful treatment strategy. Patient expectations, however, have increased; if not properly guided by surgeons, at present, patients expect next to pain-free restoration of the joint and a fast return to work and sports. While the revision rates after THA also increased in younger patients, knowledge on musculoskeletal loads still remains sparse, and the current recommendations on postoperative rehabilitation are based on expert opinions only. The aim of this study was to unravel biomechanical contact conditions in “working age” (<60 years, 53.5 ± 3.0 years) and “retirement age” (>60 years, 67.7 ± 8.6 years) patients during activities recommended post-THA. We hypothesized that working age patients would show substantially increased hip contact loads compared to older patients. The *in vivo* joint contact force (*F*_res_) and torsion torque (*M*_tors_), reflecting the main contact load situation, experienced during activities of daily living and sports activities were measured in a unique group of 16 patients with instrumented THA. We summarized patient activities and sports recommendations after THA mentioned within the literature using PubMed (without claim of completeness). The measurements showed that younger working age patients experienced significant (*p* = 0.050) increased *M*_tors_ (21.52 ± 9.11 Nm) than older retirement age patients (13.99 ± 7.89 Nm) by walking. Bowling, as a recommended low-impact sport, was associated with *F*_res_ of up to 5436 N and *M*_tors_ of up to 108 Nm in the working age group, which were higher than the *F*_res_ (5276 N) and *M*_tors_ (71 Nm) during high-impact soccer. Based on our results, age was proven to be a discriminator in joint loading, with working age patients presenting with increased loads compared to retirement age patients, already during daily activities. The current patient recommendations have led to further increased joint loadings. If THA cannot be delayed in a patient, we propose counselling patients on a carefully considered return to sports, focusing on low-impact activities, as indicated hereby. The findings from this work illustrate the need to provide critical feedback to patient expectations when returning to work and sports activities. Patients returning to more intensive sports activities should be carefully monitored and advised to avoid as much overloading as possible.

## 1 Introduction

The “operation of the century”—total hip arthroplasty (THA) (Learmonth et al., 2007)—has been demonstrated to be extremely effective for decades (Jenkins et al., 2013). THA provides a reliable solution for one of the most common disabling diseases in humans: end-stage osteoarthritis (OA) (Woolf et al., 2003; Kurtz, 2007; Pabinger and Geissler, 2014). The growing number of OA patients, their rising expectations, and an ever-growing number of younger patients seeking this surgery explain the further predicted increase in the annual number of THA surgeries (Kurtz et al., 2009; Bashinskaya et al., 2012; Nemes et al., 2014; Culliford et al., 2015; Pilz et al., 2018; Sloan et al., 2018). The incidence rate growth ranges from 25% (Pilz et al., 2018) to 132% (Sloan et al., 2018), with a 7-fold growth rate for patients <64 years (Pabinger and Geissler, 2014).

Despite the overall increase in numbers and younger patients, the lifetime of a functional hip joint arthroplasty has remained at 15–20 years over the last decades (Junnila et al., 2016; Swarup et al., 2018). Originally, THA was implemented to reduce acute pain and allow painless mobility in daily life in older patients with degenerated joints (Wright et al., 1994). For these patients, long-lasting success rates have been realized. Due to the good functional THA lifetime, new materials and design, the restriction to offer THA to older patients has been dropped and more and more younger patients now seek such end-stage therapy. At the same time, a change in work and leisure behavior took place: less physically hard work, reduction in working hours, and more attention to sports and recreational activities. In addition, patient expectations have dramatically changed over the last decades. Beyond pain reduction, the present expectations include returning not only to full functional and recreational activities (Healy et al., 2008; Mancuso et al., 2009) in daily life but also to work and sport (Hoorntje et al., 2018), or eventually to start with new activities (Schmidutz et al., 2012).

Despite all these changes, aseptic loosening remains the most common failure mode in primary THA (Ben-Shlomo et al., 2019; Burke et al., 2019; Australian Orthopaedic Association National Joint Replacement Registry (AOANJRR) 2019), especially in younger patients (Ben-Shlomo et al., 2019; Kuijpers et al., 2020). The growing number of younger compared to older patients (Australian Orthopaedic Association National Joint Replacement Registry (AOANJRR), 2019; Swedish Hip Arthroplasty Register, 2019; Norwegian National Advisory Unit Report on Arthroplasty and Hip Fractures, 2019; Danish Hip Arthroplasty Register, 2020; Canadian Institute for Health Information, 2019; Dutch Arthroplasty Register (LROI), 2019; Ben-Shlomo et al., 2019; Finnish Arthroplasty Register for National Institute of Health and Welfare, 2020) with inferior outcomes has caused an unavoidable increase in the number of THA revisions (Bayliss et al., 2017; Rajaee et al., 2018). With each additional revision surgery, the risk of implant-associated infections increases threefold (Swedish Hip Arthroplasty Register (2019); Kuijpers et al., 2020).

With the increasing number of younger patients (Pabinger and Geissler, 2014; Pilz et al., 2018; Sloan et al., 2018) seeking THA, their increased expectations for joint function and return to activity (Healy et al., 2008; Mancuso et al., 2009), their longer life expectancy (Cutler et al., 2006; Roser, 2019), and the increased economic benefit of performing such surgery, the overarching success that THA has seen in previous years may be endangered. Thus, the basic biomechanical conditions of increased joint loads in younger THA patients make serious considerations of a well-balanced recovery and expectation management essential for its long-term success.

Using a unique worldwide group of patients with telemetric THA that allows measuring *in vivo* hip contact loads (Damm et al., 2010) and comparing against recommendations on postoperative activities (Schneider et al., 2006; Bohannon, 2007; Tudor-Locke et al., 2008; Koenen et al., 2014; Bergmann et al., 2016; Oehler et al., 2016; Hoorntje et al., 2018), we hypothesized that, across a patient cohort, age is a discriminator for THA joint contact loads with younger patients showing higher loads.

## 2 Materials and Methods

### 2.1 Patients With Telemetric Total Hip Arthroplasty

**FIGURE 1:**
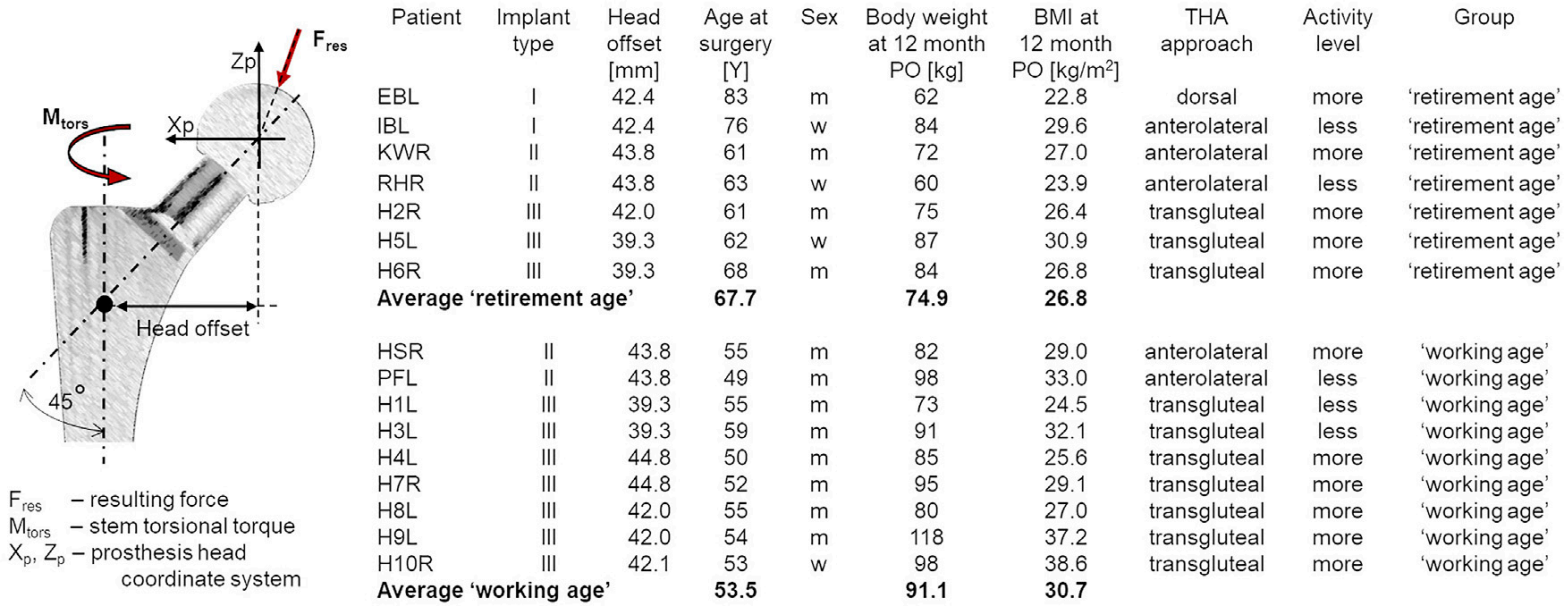
Coordinate system of the hip implant and the *in vivo* loading as the resultant contact force (*F*_res_) and torsional torque (*M*_tors_) and details of the patients wearing telemetric implants and their group distribution. Implant types: *I*—hip implant with one 4-channel transmitter technology (Bergmann et al., 1988) (since 1988); *II*—hip implant with two 8-channel transmitter technology (Graichen et al., 1999) (since 1997); and *III*—hip implant with one 9-channel transmitter technology (Graichen et al., 2007) (since April 2010).

*In vivo* loading data for THA during activities of daily living (ADL), sports, and work are rare (Bergmann et al., 1998; Bergmann et al., 2010; Bergmann et al., 2016). To identify the *in vivo* loads in THA, we used a previously described technology (Bergmann et al., 1988; Bergmann et al., 1993; Graichen et al., 1999; Damm et al., 2010; Bergmann et al., 2016; Georg Bergmann et al., 2018b). Sixteen patients with end-stage OA received between 1988 and 2013 instrumented hip implants with three different telemetric data transmission capabilities (implant types I, II, and III; Figure 1). The centrum–collum–diaphyseal (CCD) angle was 135° in all patients (Figure 1).

We used telemetric data collected in previous studies since 1990 in our well-established internal archive of *in vivo* data without new measurements. Some of the *in vivo* data have been published earlier (Bergmann et al., 2016; Damm et al., 2017; Kutzner et al., 2017). The studies were approved by our ethics committee and registered at the German Clinical Trials Register (DRKS00000563). All patients gave written informed consent prior to participation in this study and to have their images published.

Based on a subjective assessment of activity level, all patients were considered more or less active than the average activity level of the patients in the group (Figure 1). There were no changes in the activity level pre- and postoperatively.

The patients were divided into “working age” (<60 years, 53.6 ± 3.0 years) and “retirement age” (>60 years, 67.7 ± 8.6 years) groups based on their age at implantation and the lowest limit of effective retirement age in OECD (Organisation for Economic Co-operation and Development) countries. According to the *t-*test, there was a significant difference in age at implantation between the two groups [*t*(7.1) = 4.15, *p* = .004, 95% CI = 6.17–22.20], with Cohen’s effect size *d*_s_ = 2.32. The primary aim of THA in all 16 patients was pain reduction without expectation in return to sport (RTS). Two retirement age and 7 working age patients returned to work within 24 months postoperatively.

The resultant *in vivo* joint contact force (*F*_res_) acting on the implant head and the torsion torque relative to the stem (*M*_tors_), reflecting the main contact load situation at the stem–bone interface, were analyzed. *F*_res_ represents the sum of forces crossing from the acetabulum to the proximal femur *via* the head of the implant, and *M*_tors_ indicates the torsion torque acting around the implant stem (Figure 1). Notably, *M*_tors_ is derived from forces measured in the prosthesis coordinate system:

$$M_{\mathrm{tors}}=F_{\mathrm{ypr}}*L_{x}+M_{\mathrm{zpr}},$$

where *F*_*ypr*_ is the force in the anterior–posterior directions (in Newton), *M*_*zpr*_ is moment in the mediolateral direction (in Newton*meter), and *L*_*x*_ is the implant-specific head offset (in meters) (Figure 1).

Since the measured contact force and torsional torque values were compared with ISO norms to estimate whether the *in vivo* loads were of high, intermediate, or low impact, all values were considered in Newton and Newton*meter, respectively.

### 2.2 Registry Data, Self-Reported Return to Sport, and Return to Work

In order to analyze the changes in the activities and expectations of patients after THA, sports recommendations after THA, and the THA failure rates by age group in the last 30 years, several arthroplasty registries and studies to self-reported return to sport (RTS) and return to work (RTW) as the studies on sports recommendation after THA were analyzed. All respective studies were extracted from the PubMed and ScienceDirect databases. Despite a thorough search, we do not claim to have reviewed all of the published literature.

This overview summarized THA revision rate data from 10 registries (Ben-Shlomo et al., 2019; Australian Orthopaedic Association National Joint Replacement Registry (AOANJRR), 2019; Canadian Institute for Health Information, 2019; Dutch Arthroplasty Register (LROI), 2019; Norwegian National Advisory Unit Report on Arthoplasty and Hip Fractures, 2019; Danish Hip Arthroplasty Register, 2020; Finnish Arthroplasty Register for National Institute of Health and Welfare, 2020) (Supplementary Material S1), 10 studies on recommendations regarding sports after THA (McGrory et al., 1995; Healy et al., 2001; Franke et al., 2006; Klein et al., 2007; Healy et al., 2008; Swanson et al., 2009; Laursen et al., 2014; Bradley et al., 2017; Meester et al., 2018; Vu-Han et al., 2020) (Supplementary Material S2), 17 studies on RTS (Dubs et al., 1983; Mont et al., 1999; Hatterji et al., 2004; Huch et al., 2005; Suckel and Best, 2006; Arbuthnot et al., 2007; Schmidutz et al., 2012; Lefevre et al., 2013; Abe et al., 2014; Raguet et al., 2015; Innmann et al., 2016; Piccolo et al., 2016; Karampinas et al., 2017; Hara et al., 2018; Batailler et al., 2019; Jassim et al., 2019; Ortmaier et al., 2019), and 19 on RTW (Danielsson, 1965; Nevitt et al., 1984; Johnsson and Persson, 1986; Visuri et al., 1987; Suarez et al., 1996; Mobasheri et al., 2006; Bohm, 2010; Nunley et al., 2011; Buchau et al., 2013; Clyde et al., 2013; Sankar et al., 2013; Truszczyńska et al., 2014; Kleim et al., 2015; Leichtenberg et al., 2016; Pop et al., 2016; Tilbury et al., 2016; Drobniewski et al., 2017; Boersma et al., 2019; Laasik et al., 2019) (Supplementary Material S3).

### 2.3 Sport Recommendation Evidence

Expert sports recommendations were not uniform in the published literature. Different terms and numbers of intermediate levels between the recommended or allowed and non-recommended sports were used.

To provide an overview of experts’ sports recommendations, we introduced “sport recommendation evidence” (SRE) with five levels: SRE 1 for “allow”/“allowed”/“recommended”/“unlimited”; SRE 2 for “allowed with experience”/“experience”; SRE 3 for “occasional”/“intermediate”/“depends”; SRE 4 for “no conclusion”/“undecided”/“no advice”; and SRE 5 for “not recommended”/“not allowed”/“discouraged.” For known percentages of expert opinions on certain sports, we calculated the SRE as a weighted average (Supplementary Material S2).

### 2.4 Statistical Analyses of *In Vivo* Data

All evaluations were performed in R software (version 4.0.2) (R Core Team, 2021) in RStudio IDE (RStudio, Boston, MA, United States). We used *t*-tests, Spearman’s rank correlation coefficient (*r*_s_), and the Kolmogoroff–Smirnov test for group differences, which were evaluated using the stats package (R software).

For the analysis of covariance (ANCOVA), the R script as proposed in Wollschläger (2017) was applied. The 95% confidence interval (CI), *p*-value, and effect size were reported. A *p* < .05 was considered significant.

## 3 Results

### 3.1 Relevance of Body Weight and Age in Maximal Total Hip Arthroplasty Contact Forces and Torsional Torques

**FIGURE 2:**
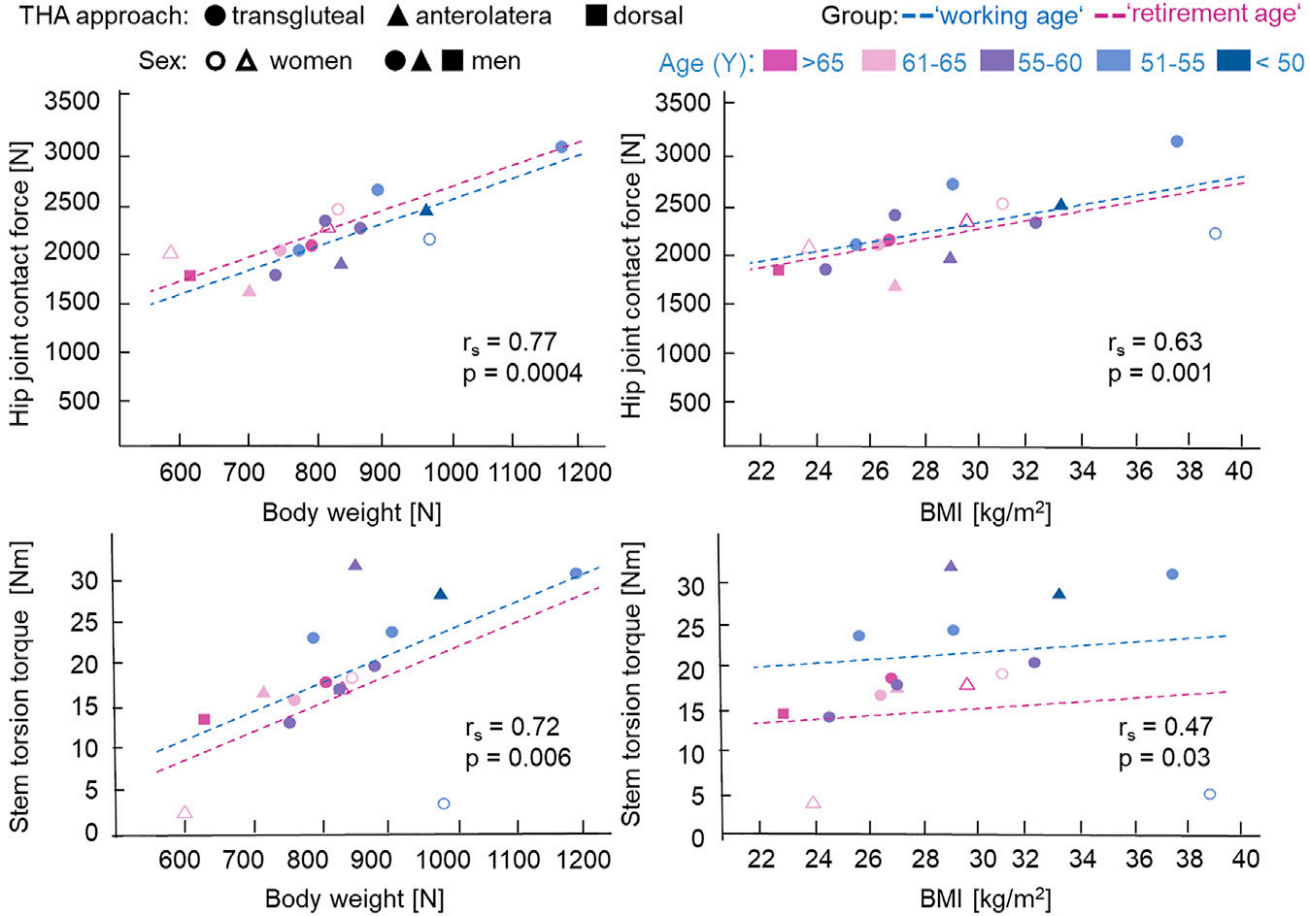
Influence of body weight (BW), body mass index (BMI), and age on the maximal hip joint contact force and stem torsion torque during normal walking with self-selected comfortable speed. For total hip arthroplasty (THA) approach, *circle* denotes transqluteal, *triangle* denotes anterolateral, and *rectangle* denotes dorsal. *Unfilled circle* or *triangle* represents women, while *filled circle*, *triangle*, or *rectangle* represents men. Age at implantation is shown from *magenta* (>65 years) to *blue* (<50 years) in 5-year steps. *r*_s_ is the Spearman’s rank correlation coefficient (one-tailed) for all patients (working age and retirement age groups) with *p*-values. Analysis of covariance (ANCOVA) with separated regression lines by groups: *dashed blue lines* for working age (<60 years) and *dashed magenta lines* for retirement age (>60 years).

**FIGURE 3:**
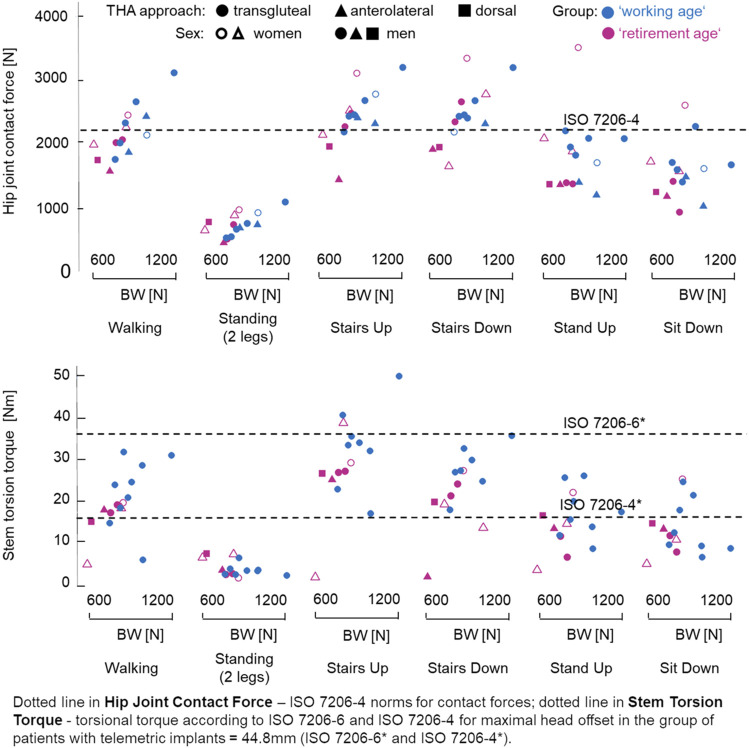
Age-related differences in the maximal hip joint contact force, *F*_res_ (in newton), and maximal torsion torque, *M*_tors_ (in Newton*meter), during various activities of daily living (ADL). For total hip arthroplasty (THA) approach, *circle* denotes transqluteal, *triangle* denotes anterolateral, and *rectangle* denotes dorsal. *Empty circle* or *triangle* represents women, while *filled circle*, *triangle*, or *rectangle* represents men. For group by age at implantation, the retirement age group (>60 years) is shown as magenta and the working age group (<60 years) shown as blue.

Our data showed no relationship between the THA approach (dorsal, transgluteal, or anterolateral) or sex and implant loading (Figures 2, 3).

The younger working age patient group had, on average, a higher body weight (BW)/body mass index (BMI) (91.1 ± 13.3 kg, BMI = 30.7 ± 4.9) than the older retirement age group (74.9 ± 10.9 kg, BMI = 26.8 ± 2.9). The *t-*test indicated a significant difference in BW/BMI between both groups [BW: *t*(13.9) = 2.69, *p* = 0.018, 95% CI = 3.31–29.20; BMI: *t*(13.1) = 1.98, *p* = .069, 95% CI = −.35 to 8.16)], with a strong Cohen’s effect size (BW: *d*_s_ = 1.32; BMI: *d*_s_ = .93).

Spearman’s rank correlation test showed a significant positive correlation between the maximal joint contact force, *F*_res_, and BW/BMI (BW: *r*_s_ = .77, *p* < .001; BMI: *r*_s_ = .63, *p* = .001) and between the maximal stem torsion torque, *M*_tors_, and BW/BMI (BW: *r*_s_ = .72, *p* = .006; BMI: *r*_s_ = .47, *p* = .03) during normal walking with self-selected speed for all our patients (Figure 2), which meant a significant strong (for *r*_s_ = .77, .63, or .72) to moderate (for *r*_s_ = .47) correlation.

Since the data points in Figure 2 were color-coded gradually from magenta for age >65 years to blue for age <50 years, it can be observed that the working age group tended to have higher *F*_res_ and *M*_tors_. The influence of age on *F*_res_ and *M*_tors_ was assessed using ANCOVA with BW/BMI as the confounder. ANCOVA showed a significant dependency of *F*_res_ [*F*(1, 7, 9) = 5.07, *p* = .042, with partial *η*_p_^2^ as effect size = .064] and a lower dependency of *M*_tors_ [*F*(1, 7, 9) = 3.60, *p* = .080, with effect size *η*_p_^2^ = .016] on age group at walking. The regression lines for both groups are shown in Figure 2 and demonstrated a tendency of higher contact force and torsion torque values for the working age group; only for *F*_res_ = *f*(BW) did the regression line for the retirement age group lie higher than that for the working age group.

### 3.2 *In Vivo* Joint Loads During Activities of Daily Living

Since the revision rates in younger patients were higher and there were indications of age-related differences in the movement patterns due to changes in muscular coordination, it is necessary to consider differences between these groups. For comparison, the 12-month postoperative *F*_res_ and *M*_tors_ for each patient and ADL were used.

While the maximal *F*_res_ in the working age group (2,327 ± 441 N) tended to be higher, but demonstrated no significant differences compared to the retirement age group (2,057 ± 291 N) by walking at self-selected speed, comparison of the maximal *M*_tors_ between the groups resulted in significantly higher torsional torque values in the working age group (21.52 ± 9.11 Nm) than in the retirement age group (13.99 ± 7.89 Nm) for this activity. The Kolmogorov–Smirnov test for maximal *M*_tors_ revealed significant differences (*D* = .625, *p* = .050).

While there were trends toward higher maximal contact force and torsion torque values in the working age group across all activities (Figure 3), no significant differences were identified in all other considered ADL. However, the force values during ADL were higher than that of the test standard for the implant stem (ISO7206-4) and lower than that for the neck (ISO7206-6). According to standards ISO7206-6 and ISO7206-4, the maximal individual test *M*_tors_ values in our patient group were 37.4 and 16 Nm, respectively. Almost all ADL in our measurements showed values higher than 16 Nm and lower than 37.4 Nm. However, the *M*_tors_ values were partly higher than those of the test standards during stair climbing. Patients with lower BW/BMI showed, in some cases, higher (stairs down/stand up) or similar (stairs up) *F*_res_ and *M*_tors_ (stairs up/down).

### 3.3 Changes in Patient Age and Expectations

Data on the cumulative revision rates from arthroplasty registries for different age groups, for both men and women, were compared (Ben-Shlomo et al., 2019; Australian Orthopaedic Association National Joint Replacement Registry (AOANJRR), 2019; Canadian Institute for Health Information, 2019; Danish Hip Arthroplasty Register, 2020; Finnish Arthroplasty Register for National Institute of Health and Welfare, 2020; Dutch Arthroplasty Register (LROI), 2019; Norwegian National Advisory Unit Report on Arthoplasty and Hip Fractures, 2019). The revision rates at 1 year postoperative time for patients <55 years (.92%–2.1% for males and .92%–2.0% for females) were similar or slightly lower than those for patients >75 years (.98%–2.6% for males and .71%–2.03% for females). At 5 years postoperative time, the revision rates for patients <55 years (3.33%–5.5% for males and 3.11%–7.0% for females) were higher than those for patients >75 years (2.05%–4.08% for males and 1.48%–4.33% for females). At 20–25 years postoperatively, the cumulative revision rates for younger patients (<55 years) (23.7%–44.3% for males and 24.5%–51% for females) were significantly higher than those for older patients (>75 years) (13.2%–25.6% for males and 9.1%–17.5% for females) (for more details, see Supplementary Material S1).

The age of THA patients was analyzed in some studies (Kurtz et al., 2009; BQS—Bundesgeschäftsstelle Qualitätssicherung gGmbH, 2009; IQTIG–Institut für Qualitätssicherung und Transparenz im Gesundheitswesen, 2018). These studies revealed that every sixth to fifth THA patient is younger than 60 years. Five studies (Bashinskaya et al., 2012; Nemes et al., 2014; Culliford et al., 2015; Pilz et al., 2018; Sloan et al., 2018) have shown a tendency toward younger patients.

Several studies analyzed the expectations of patients after THA (Wright et al., 1994; Mancuso et al., 2009; Ghomrawi et al., 2011; Hepinstall et al., 2011; Judge et al., 2011; Scott and Biant, 2012; Koenen et al., 2014; Mancuso et al., 2017; Jassim et al., 2019). The studies of Koenen et al., (2014), Wright et al., (1994), and Mancuso et al., (2009) showed that higher expectations in THA were related to younger age (Gandhi et al., 2010; Scott and Biant, 2012). Two studies reported age-independent high expectations (Hepinstall et al., 2011; Koenen et al., 2014). At least six studies reported high patient expectations of full recovery in ADL and sports (Mancuso et al., 2009; Ghomrawi et al., 2011; Scott and Biant, 2012; Koenen et al., 2014; Mancuso et al., 2017; Jassim et al., 2019). Oehler et al., (2016) showed the overall increasing level of activities of the THA patients in their study.

### 3.4 Sports-Related Recommendations After Total Hip Arthroplasty

Sports after THA is a significant health factor for patients. Based on 10 studies published between 1995 and 2020 (McGroryet al., 1995; Healy et al., 2001; Franke et al., 2006; Klein et al., 2007; Healy et al., 2008; Swanson et al., 2009; Laursen et al., 2014; Bradley et al., 2017; Meester et al., 2018; Vu-Han et al., 2020), we compiled an overview of sports recommendations after THA over the past 20–30 years (Supplementary Material S2).

The sports activity recommendations in all 10 studies have been developed on the basis of expert opinions (Healy et al., 2001; Klein et al., 2007; Healy et al., 2008; Reeves et al., 2009) and divided sports into three severity levels according to the assumed load levels (Vail et al., 1996; Schmidutz et al., 2012): low, intermediate, and high impact for low, intermediate, and high expected loads, respectively. There was no uniform opinion on sports classifications (Klein et al., 2007; Schmidutz et al., 2012; Hara et al., 2018) with

– Cross-country skiing was classified as “potentially low” by Klein et al. (2007) and as “intermediate” by Schmidutz et al. (2012).
– Aerobics was classified as “intermediate” by Hara et al. (2018), while Klein et al. (2007) distinguished between low-impact aerobics, which was classified as “intermediate impact,” and high-impact aerobics, which was classified as “high impact.”

The number and type of sports activities considered also differed between studies. McGrory et al. (1995) considered 28 activities, Healy et al. (2008) considered 36, and the study by Meester et al. (2018) considered 41 sports activities with recommendations for patients >65 and <65 years.

Expert recommendations varied between “allow”/“allowed”/“recommended”/“unlimited”/“without limitation” and “not recommended”/“not allowed”/“discouraged” with 1–3 steps in between. To allow a comparison of expert sports recommendations, we introduced SRE with values between SRE 1 for “allow”/“allowed”/“recommended”/“unlimited”/“without limitation” and SRE 5 for “not recommended”/“not allowed”/“discouraged” (see *Materials and Methods* and Supplementary Material S2). A comparison between the different recommendations indicated changes in expert opinions within the last 20–30 years. A change within the SRE values can be observed in multiple cases, as in the following:

– For hiking: from SRE 2 (“allowed with experience”/“experience”/“with training”) in McGrory et al. (1995) to SRE 1 (“allow”/“allowed”/“recommended”/“unlimited”/“without limitation”) in Bradley et al. (2017) and Vu-Han et al. (2020)
– For tennis doubles: from SRE 3 (“occasional”/“intermediate”/“depends”) (McGrory et al., 1995) to SRE 2 (Healy et al., 2008; Meester et al., 2018)
– For dancing (ballet und square/jazz): from SRE 4 (“no conclusion”/“undecided”/“no advice”) (McGrory et al., 1995; Healy et al., 2001) to SRE 1(Meester et al., 2018; Vu-Han et al., 2020)
– For jogging: from SRE 5 (“not recommended”/“not allowed”/“discouraged”) (Healy et al., 2001; Klein et al., 2007) to SRE 2 (Laursen et al., 2014; Vu-Han et al., 2020)

In Supplementary Material S2, more data collected on sports activity recommendations after THA can be found.

### 3.5 Self-Reported Return to Sports After Total Hip Arthroplasty

Data from 17 studies on RTS sorted by impact were summarized (Supplementary Materials S2 and S3). Two of the 17 studies reported RTS before 2000 postoperatively relative to preoperatively. In the study from 1983 (Dubs et al., 1983) with 110 THA patients, 102% of the patients returned to low-impact sports (>100% means that patients had not performed sports before THA and began sports participation after), 50% returned to intermediate-impact, and 40% to high-impact sports. The studies after 2000 with up to 420 THA patients showed an RTS of 90%–180% for low-impact, 40%–140% for intermediate-impact, and 10%–100% for high-impact sports activities.

### 3.6 Self-Reported Return to Work After Total Hip Arthroplasty

From the 19 studies on self-reported RTW for patients <65 years at the time of surgery, five studies were published before 2000 and reported RTW between 25% (Suarez et al., 1996) and 68% (Nevitt et al., 1984). The other 14 studies were published after 2000 and reported RTW between 59% (Truszczyńska et al., 2014) and 96%(Mobasheri et al., 2006). The summarized RTW value for five studies before 2000 was at 43% and for the 14 studies after 2000 was at 90%.

### 3.7 *In Vivo* Loads During Sporting Activities

**FIGURE 4:**
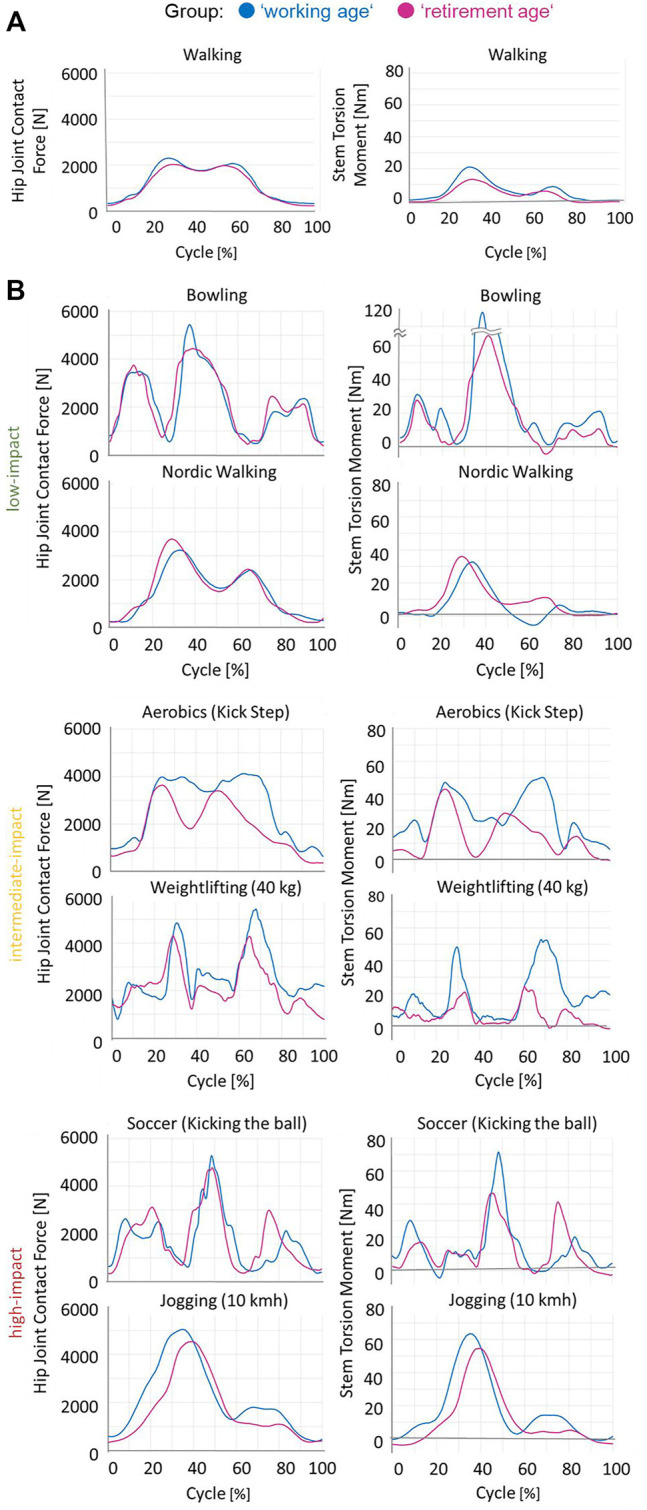
**(A)** Mean *in vivo* hip joint contact force and stem torsion torque curves during walking (ADL, Figure 3). **(B)** Examples of the *in vivo* measured hip joint contact force and torsion torque values for some low-, intermediate-, and high-impact sporting activities, as classified in Supplementary Material S2, for one patient per group, working age (*blue*) and retirement age (*magenta*).

To obtain an impression of the magnitude of *F*_res_ and *M*_tors_, we present in Figure 4 the results of our first *in vivo* data collected during activities that were ranked according to the above classifications into low-, intermediate-, and high-impact sports levels.

In the <60 years group, not only the higher maximum but also the average contact force and torsion torque values across the entire movement cycle were increased, with clear differences between age groups:

–Additional “in-betweens–low points” in *F*_res_ and *M*_tors_ in aerobic exercise and
–Significantly “flatter” curve of the *M*_tors_ in weightlifting in elderly patients.

**FIGURE 5:**
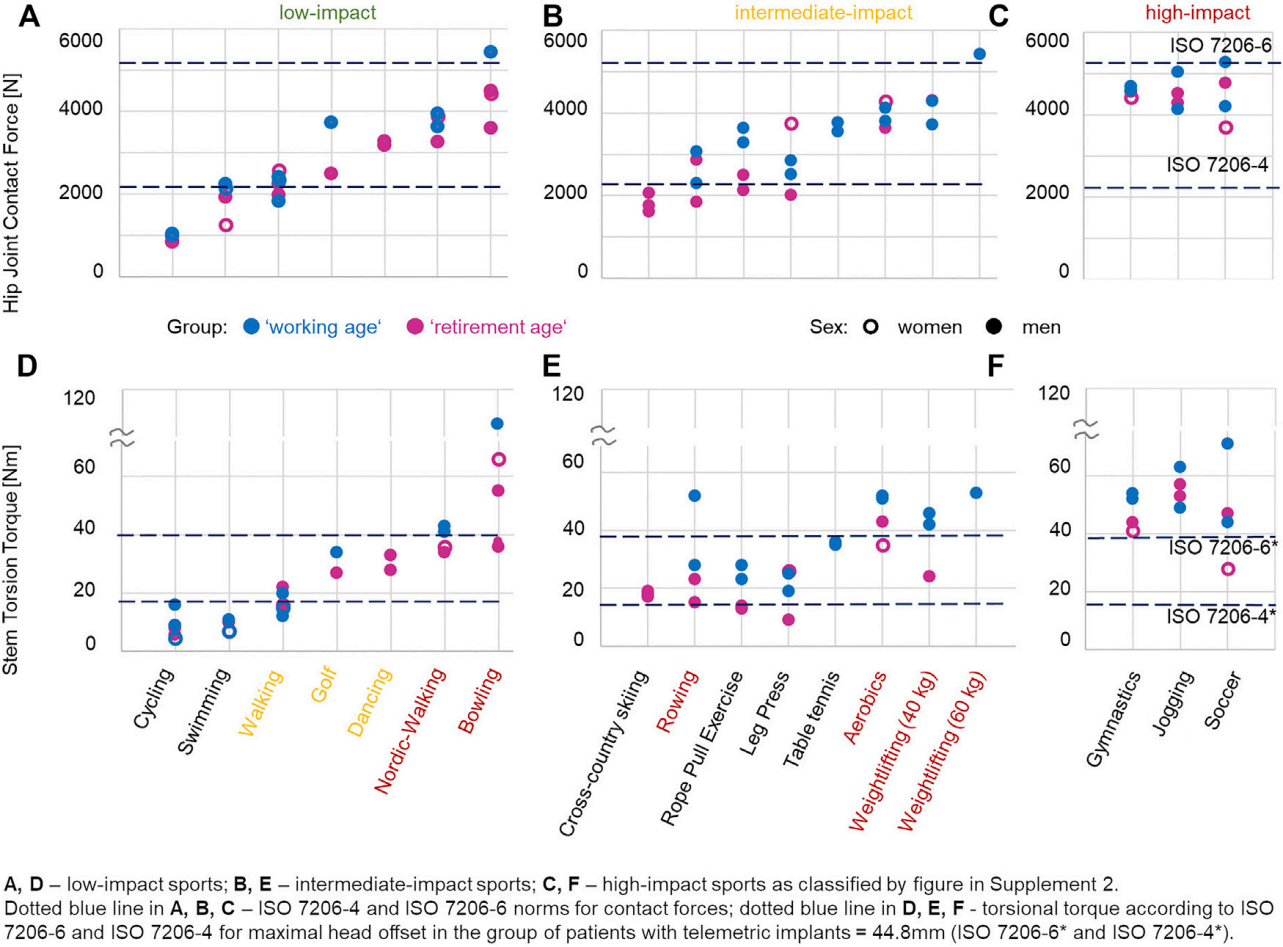
Examples of the *in vivo* measured hip joint contact force **(A–C)** and torsion torque **(D–F)** values for some sporting activities. One to three examples per group, retirement age (*magenta*) or working age (*blue*), are drawn. *Filled circle* and *empty circle* denote men and women, respectively. *Red* low- and intermediate-impact activities show high-impact contact force and torsion torque values. *Yellow* low-impact activities show intermediate-impact contact force and torsion torque values.

There were a limited number of measurements available at present, and the maximum estimated *M*_tors_ and *F*_res_ values were determined for two subjects per group (Figure 5). All sports activities were performed by more active patients (Figure 1) who had undergone a transgluteal THA approach, except golf (anterolateral) and cross-country skiing (dorsal).

High loads (*F*_res_ and *M*_tors_) occurred in both high-impact (*F*_res_ = 5276 N, *M*_tors_ = 71 Nm at soccer) and low-impact (*F*_res_ = 5436 N, *M*_tors_ = 108 Nm at bowling) sports. Therefore, patients in the working age group tended to have higher *F*_res_ and *M*_tors_. In bowling, rowing, and soccer, patients from the working age group with BMI of 25.6 (H4L) showed higher *F*_res_ and *M*_tors_ than patients with BMI of 26.4 (H2R), 26.5 (H6R), or 30.9 (H5L).

## 4 Discussion

THA has shown a documented track record of being a successful therapy for late-stage OA so far and has been proven to be an instant solution for patients suffering from pain. However, various studies have confirmed that today’s THA patients are more active than those who underwent THA in the 1980s–1990s (Bohannon, 2007; Naal and Impellizzeri, 2010; Hoorntje et al., 2018). They are more active in ADL and frequently return to work after surgery, but they also return to sports and have high expectations for the endurance and survival of their joint replacement.

In this study, the differences in the contact force and stem torsion torque values between working age and retirement age THA patients were explored in a small but unique group of patients with telemetric THA. The small number of patients is a limitation of this study. However, this unique worldwide group provided an insight into tendencies in THA loading conditions for younger and older THA patients.

A high BMI is a significant risk factor for several diseases (NCD Risk Factor Collaboration, 2016). The rate of obese THA patients with BMI > 30 and significantly higher complication rates (Jakubowitz et al., 2009; Zhang et al., 2012) increased (Culliford et al., 2015; Buirs et al., 2016). Several studies showed age-related changes in movement patterns and suggested that these were due to altered muscular coordination and performance (Hortobágyi et al., 2011; Novak and Brouwer, 2011; Chiu et al., 2015). It was discussed that this should ultimately also lead to altered loads in the large joints of the lower extremity (Hortobágyi et al., 2011).

Our patient groups reflected the current situation in the population (Finucane et al., 2011; Hales et al., 2017): the working age group had, on average, a higher BW/BMI (91.1 kg, BMI = 30.7) than the retirement age group (74.9 kg, BMI = 26.8). However, our measurements revealed that BW was an essential, but not the only, factor in the THA loading conditions: in bowling, rowing, and soccer, as in some ADL (stairs down/up and stand up), patients with low BMI showed the highest loading.

Our measurements confirmed the intuitive assumption that working age patients experience higher contact forces and torsion torques than do retirement age patients. This statement refers to ADL (Figure 3) and the aforementioned examples of sport activities (Figures 4, 5). The loads—even during ADL, such as stair climbing *M*_tors_—may exceed the thresholds defined in ISO7206-6 for preclinical testing for THA in younger patients.

Other potential influencing parameters, such as a change in lean muscle volume of the gluteal muscles (Damm et al., 2018; Heller et al., 2001), could affect the contact force and torsion torque values [like a reversed effect for *F*_res_ = *f*(BW)] (Figure 2), but could not be controlled in this study and were not taken into account here.

Various registries revealed higher revision rates for younger patients than for older patients (Ben-Shlomo et al., 2019; Australian Orthopaedic Association National Joint Replacement Registry (AOANJRR) 2019; Swedish Hip Arthroplasty Register, 2019; Norwegian National Advisory Unit Report on Arthoplasty and Hip Fractures, 2019; Danish Hip Arthroplasty Register, 2020; Canadian Institute for Health Information, 2019;Dutch Arthroplasty Register (LROI) 2019; Finnish Arthroplasty Register for National Institute of Health and Welfare, 2020) (Supplementary Material S1) for both males and females, especially from 3 to 5 years postoperatively. Other studies have shown that the higher expectations in THA (Wright et al., 1994; Mancuso et al., 2009; Koenen et al., 2014) were related to younger age (Gandhi et al., 2010; Scott and Biant, 2012) and that every sixth to fifth patient was younger than 60 years (BQS—Bundesgeschäftsstelle Qualitätssicherung gGmbH, 2009; Kurtz et al., 2009; IQTIG—Institut für Qualitätssicherung und Transparenz im Gesundheitswesen, 2018), with a continuous trend toward younger patients (Bashinskaya et al., 2012; Nemes et al., 2014; Culliford et al., 2015; Pilz et al., 2018; Sloan et al., 2018). The reported age-independent high expectations in Hepinstall et al. (2011) and Koenen et al. (2014) may be explained by the overall increasing level of activities even in patients above 60 years of age (Oehler et al., 2016).

In general, patients today come with high expectations of full recovery of daily and sporting activities (Mancuso et al., 2009; Ghomrawi et al., 2011; Scott and Biant, 2012; Koenen et al., 2014; Mancuso et al., 2017; Jassim et al., 2019). Patient expectations were characterized by a desire to maintain relevant mobility for a long-term perspective with limited acceptance of any postoperative constraints on their preoperative activity levels (Mancuso et al., 2009; Mancuso et al., 2017). In some reports, patients even associated THA with restoration of greater athletic endurance (Judge et al., 2011; Mancuso et al., 2017; Jassim et al., 2019). Fulfilling these expectations appeared to correlate well with postoperative satisfaction with THA (Scott and Biant, 2012).

Without a doubt, engaging in sports has beneficial effects on reducing the risk of cardiovascular diseases and maintaining bone quality and neuromuscular coordination (Haskell et al., 2007; Global Recommendations on Physical Activity for Health, 2012). Contrary to the original recommendation (Charnley, 1979)—which discouraged patients from anything more strenuous than walking—the current recommendations suggest RTS activity (Swanson e al., 2009; Laursen et al., 2014; Meester et al., 2018).

Due to the lack of *in vivo* data, sports activity recommendations after THA have been widely developed on an expert opinion basis (Healy e al., 2001; Klein et al., 2007; Healy et al., 2008; Reeves et al., 2009) and divided sports into low, intermediate, and high impact (Vail et al., 1996; Schmidutz et al., 2012) for low, intermediate, and high expected loads, respectively. There is no uniform opinion on sports classifications (Klein et al., 2007; Schmidutz et al., 2012; Hara et al., 2018), nor have these classifications remained unchanged over the years (McGrory et al., 1995; Healy et al., 2001; Healy et al., 2008; Meester et al., 2018). Supplementary Material S2 gives an overview of the recommendations (McGrory et al., 1995; Healy et al., 2001; Klein et al., 2007; Healy et al., 2008; Swanson et al., 2009; Laursen et al., 2014; Bradley et al., 2017; Meester et al., 2018; Vu-Han et al., 2020), without claiming to be a complete overview. It quickly becomes visible that the recommendations are not uniform and that they differ among authors: 20–25 years ago, only a few sporting activities were “recommended” (McGrory et al., 1995; Healy et al., 2008), while today, the number has substantially increased (Klein et al., 2007; Laursen et al., 2014; Bradley et al., 2017; Meester et al., 2018; Vu-Han et al., 2020). Expert opinion-based recommendations have changed over the years, from being rather “conservative” to now being more “risk-friendly.” However, the published recommendations do not refer to *in vivo* loads or clinical registry or cohort data, and it remains unclear on what scientific basis were these recommendations formulated (Klein et al., 2007; Laursen et al., 2014; Bradley et al., 2017; Meester et al., 2018; Vu-Han et al., 2020).

Only a few studies reported RTS before 2000 (Dubs et al., 1983; Mont et al., 1999). The data after 2000 showed the same trend as 3 years before (Hoorntje et al., 2018) in terms of increased sports activities. Many patients practice increasingly high-impact sports (McGrory et al., 1995; Jassim et al., 2014; Hoorntje et al., 2018; Vu-Han et al., 2020). A study in a wider population (Bonnin et al., 2018) showed that approximately 20% of patients participated in strenuous sports and that 76% were motivated.

The summarized data from 19 studies on self-reported RTW for patients <65 years at the time of surgery (Supplementary Material S3) showed that significantly fewer patients returned to work until 2000. After 2000, on average, approximately 90% returned to work, which confirmed the trend identified in systematic reviews (Kuijer et al., 2009; Tilbury et al., 2014; Hoorntje et al., 2018) and can be explained at least in part by the fact that the number of younger patients of working age has increased. Many of them switched to less heavy-duty work (Hoorntje et al., 2018), although the actual loads experienced during work were generally unknown. While we do not claim to have reviewed all of the published literature, this overview provide a survey of the changes in activity and recommended activity after THA over the past two decades.

The *in vivo* loads during sporting activities were mostly unknown, except for those activities measured in our small cohort of patients (Kutzner, et al., 2017; Damm et al., 2017; Kutzner et al., 2017; Haffer et al., 2021). However, there is evidence that joint friction differed markedly between ADL and sports activities (Georg Bergmann, et al., 2018a). It can be expected that sports will also lead to higher *F*_res_ and *M*_tors_. Therefore, it is not surprising that sport activities generally lead to experiencing higher loads. However, the levels of torsional torque were unexpected: while the maximum force levels across all activities were roughly comparable, the maximal torsional torque, *M*_tors_, measured in low-impact sports was even higher than that in some activities considered to be of high impact. Maximum *M*_tors_ was observed during bowling, previously classified (Ritter and Meding, 1987) as a low-impact sport (Supplementary Material S2).

In specific sport activities, the loads were even higher than those of the preclinical test standard loads for neck failure testing (ISO7206-6). *M*_tors_ exceeded the levels defined by both ISO standards in our patients. Only in swimming and cycling were the maximal *F*_res_ and *M*_tors_ within the preclinical test standards for THA. In the <60 years group, not only the high load maxima but also the load levels experienced across the entire cycles of various sport activities were increased compared to those in the retirement age group (Figure 4).

These measurements are only first impressions of the load patterns during sporting activities. Additional investigations of such telemetrically instrumented patients will be necessary to gain an in-depth understanding of the influence of specific sports activities on biomechanical conditions and consequences on a tissue level, such as at the implant–bone interface, in the various age groups.

Mobility and sports activity are vital for patients with THA to achieve a balanced life and to meet their expectations. Several reviews have shown a trend toward increased self-reported sport activities with increasing intensity (Schmidutz et al., 2012; Bonnin et al., 2018; Hoorntje et al., 2018). Patients performing judo (Lefevre et al., 2013) and jogging (Abe et al., 2014) and many other sports activities were not at all covered (Schmidutz et al., 2012; Bonnin et al., 2018).

With this overview, we aimed to open a debate on the current postoperative recommendations. The data on *in vivo* loads, specifically the increased torsional torque in working age patients and its further increase even in the so-called low-impact sports activities, make it necessary to revisit the current “guidelines” for RTS after THA. Our measurements suggest that only swimming and cycling are at load levels below those of ADL and can be considered “low impact” in any postoperative setting. Only such activities should be “recommended” to those patients in whom a low-impact sport appears to be necessary. In all other activities, we found load levels higher than those experienced during ADL. *In vivo* loads during work were mostly unknown, but it can be supposed that these loads may be reflected in some of the sport activities that we were able to measure, especially for the working age patients.

The calculated revision risk (Bayliss et al., 2017) for patients <60 years at primary surgery appeared to be up to 35%, resulting in younger patients also having an increased re-revision risk (Lübbeke et al., 2007; Ben-Shlomo et al., 2019), while the lifetime risk for patients >70 years was much lower (1%–6%) (Bayliss et al., 2017). With further increases in other factors, such as activity level, number of patients working after surgery, and life expectancy, it can be expected that the lifetime risk of revision for younger patients may even be higher. Consequently, the revision and re-revision numbers are expected to rise further in the next decade.

From a biomechanical perspective, THA, as the ultimate solution, should be delayed as much as possible, taking into account the consequences of OA such as pain (Vergara et al., 2011), impact on ADL (Palazzo et al., 2016; Clynes et al., 2019), mental well-being (Vina and Kwoh, 2018), and the risk of all-cause mortality (Palazzo et al., 2016; Kendzerska et al., 2018). If THA cannot be delayed in a patient, we propose reconsidering the current recommendations critically and making patients aware of the risk of potential implant loosening during full return to sports. Moreover, recommendations of sportive activities should be mainly focused on low-impact activities, as indicated here. If patients have to do heavy physical work or are nevertheless interested in intermediate-/high-impact sports, they should be aware of the implications this may have on the longevity of THA. The findings from this paper and the data from http://www.OrthoLoad.com may help guide patient expectations.

Various registries revealed higher revision rates for younger patients than for older patients. Patients today come with higher expectations, and current recommendations suggesting RTS activity showed that the contact force and torsional torque values were increased in younger (working age) compared to older (retirement age) patients for daily and sporting activities. If patients engage in intermediate- or high-impact sports despite being informed of the implications, they have to be closely monitored clinically and radiologically. Further study of the biomechanical loading in sports and work is needed.

## Data Availability

The datasets presented in this study can be found in online repositories. The names of the repository/repositories and accession number(s) can be found below: https://orthoload.com/database/.
