# Supplementary material for: Overstretching Expectations May Endanger the Success of the “Millennium Surgery”
Source: Front Bioeng Biotechnol. 2022 Feb 14;10:789629. doi: 10.3389/fbioe.2022.789629 (PMC8882767; doi:10.3389/fbioe.2022.789629)
Supplement: Supplementary file 1 [file DataSheet3.docx]

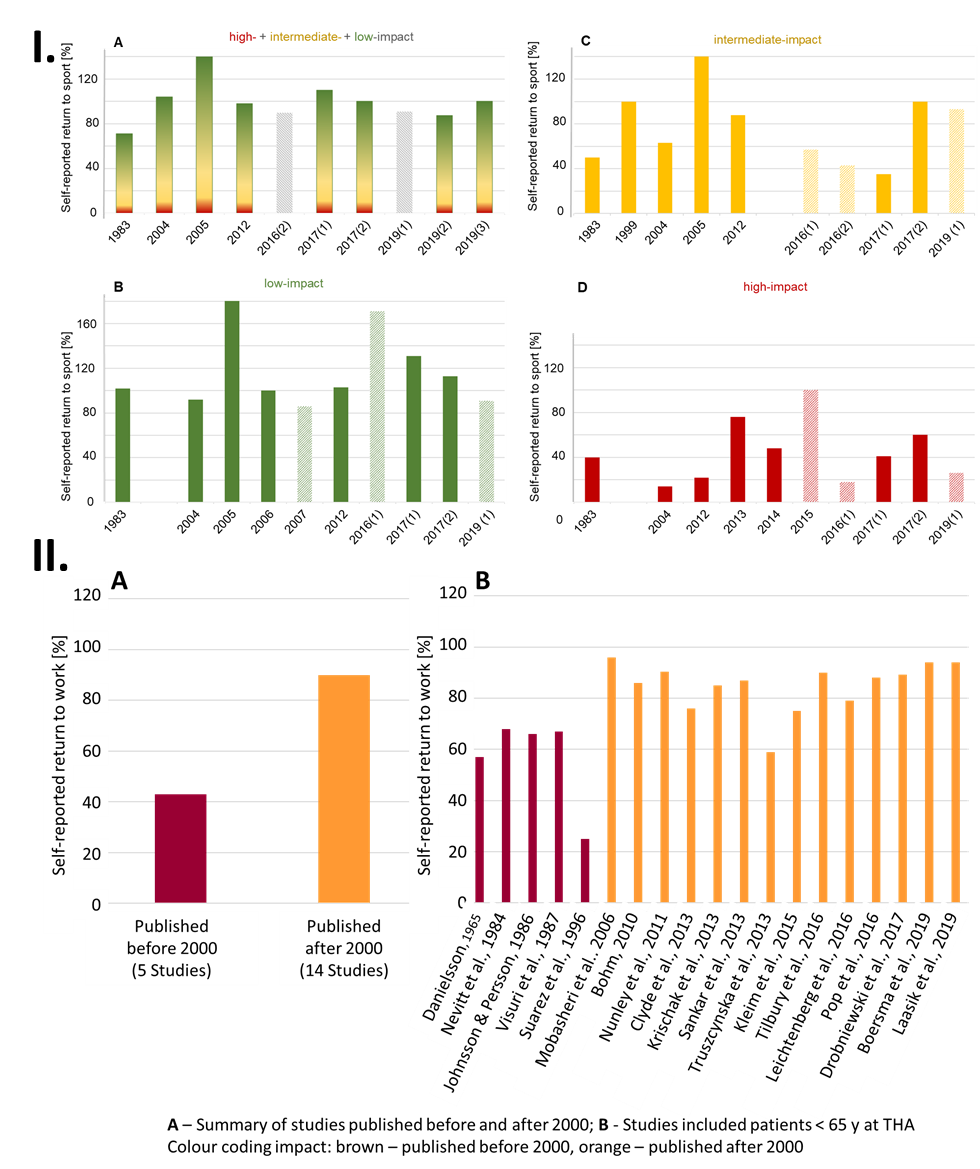
**Supplement 3.**
I. Self-reported return to sport in % (unicolored – postoperatively relative to preoperatively; dashed – postoperatively to pre-symptomatic) from 17 studies; low-, intermediate- and high-impact sports as classified in Figure (Supplement Figure 2).

Return to sports >100% means patients had not performed any sport prior to the surgery and began sport participation after.

I.-A: for low-, intermediate- and high-impact sports
I.-B: for high-impact sports
I.-C: for intermediate-impact sports
I.-D: for low-impact sports

Coding studies:

1983 - Dubs et al., 1983; 1999 – Mont et al., 1999; 2004 - Chatterji et al. 2004;
2005 - Huch et al., 2005; 2006 - Suckel and Best, 2006; 2007 - Arbuthnot et al., 2007;
2012 - Schmidutz et al., 2012; 2013 – Lefevre et al., 2013; 2014 - Abe et al., 2014;
2015 - Raguet et al., 2015; 2016(1) - Innmann et al., 2016; 2016(2) - Del Piccolo et al., 2016; 2017(1) - Hara et al., 2017; 2017(2) - Karampinas et al., 2017; 2019(1) – Ortmaier et al., 2019
2019(2) – Batailler et al., 2019; 2019(3) –Jassim et al., 2019

II. Self-reported return to work in % (postoperatively relative to preoperatively) from 19 studies
II.-A: Summary of studies published before and after 2000
II.-B: Studies included patients < 65 y at THA
Colour coding: brown – published before 2000; orange – published after 2000
