# Supplementary material for: Overstretching Expectations May Endanger the Success of the “Millennium Surgery”
Source: Front Bioeng Biotechnol. 2022 Feb 14;10:789629. doi: 10.3389/fbioe.2022.789629 (PMC8882767; doi:10.3389/fbioe.2022.789629)
Supplement: Supplementary file 2 [file DataSheet2.docx]

***
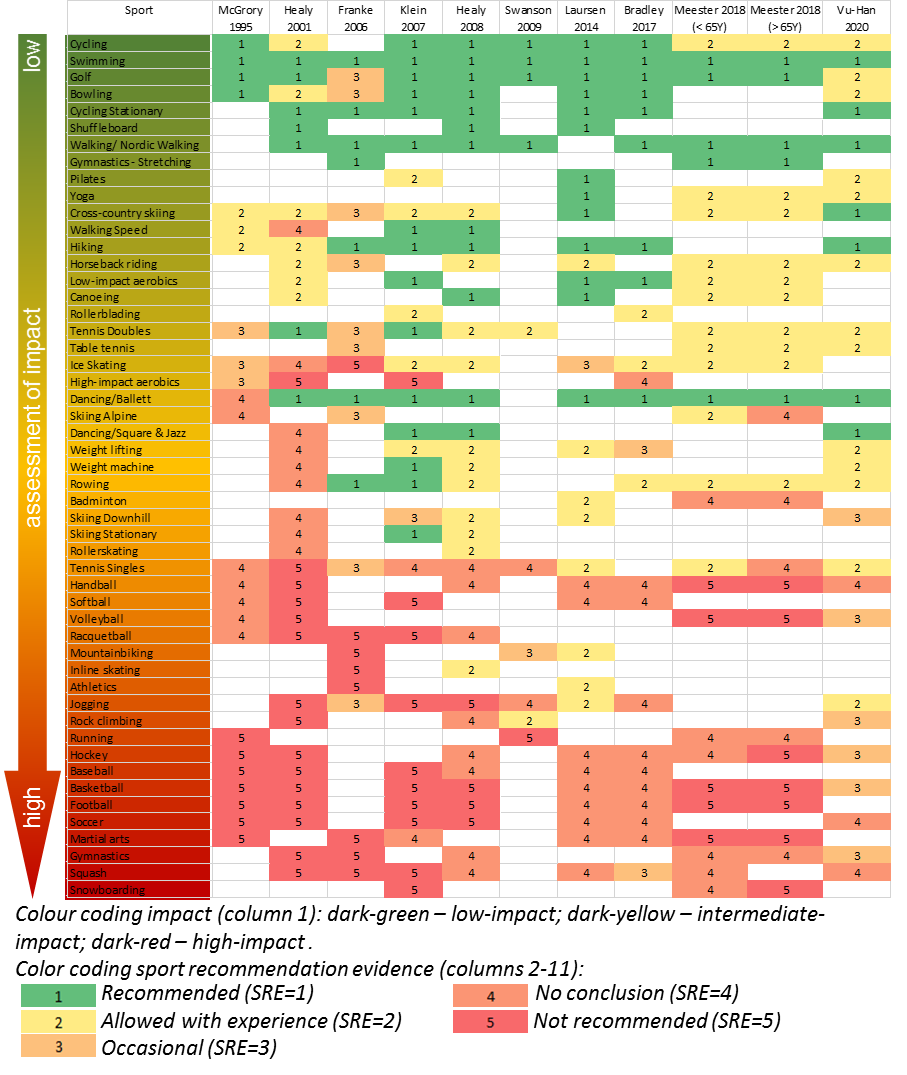
***

**Supplement 2**. Sports recommendation for THA patients in McGrory et al., 1995, Healy et al., 2001, Franke et al., 2006, Klein et al., 2007, Healy et al., 2008, Swanson et al., 2009, Laursen et al., 2014, Bradley et al., 2017, Meester et al., 2018; Vu-Han et al., 2020

To gain an overview of the similarities/discrepancies in the impact experienced during sports, we coded the impact assignment with colours from dark green (low-impact) to dark yellow to dark red (high-impact). However, there is no uniform expert opinion on the classification of different sports, so the borders of the colours are blurred.

To allow a comparison of experts' sport recommendations, ‘Sport Recommendation Evidence’ (SRE) was introduced:

SRE=1 ("Allow"/"Allowed"/"Recommended"/“Unlimited”/”Without limitation”)
SRE=2 (“Allowed with experience”/“Experience”/”With training”)
SRE=3 (“Occasional”/“Intermediate”/”Depends”)
SRE=4 (“No conclusion”/“Undecided”/“No advice”)
SRE=5 (“Not recommended”/“Not Allowed”/“Discouraged”).
SRE is calculated as a weighted average.
