# Supplementary material for: Overstretching Expectations May Endanger the Success of the “Millennium Surgery”
Source: Front Bioeng Biotechnol. 2022 Feb 14;10:789629. doi: 10.3389/fbioe.2022.789629 (PMC8882767; doi:10.3389/fbioe.2022.789629)
Supplement: Supplementary file 3 [file DataSheet1.docx]

***
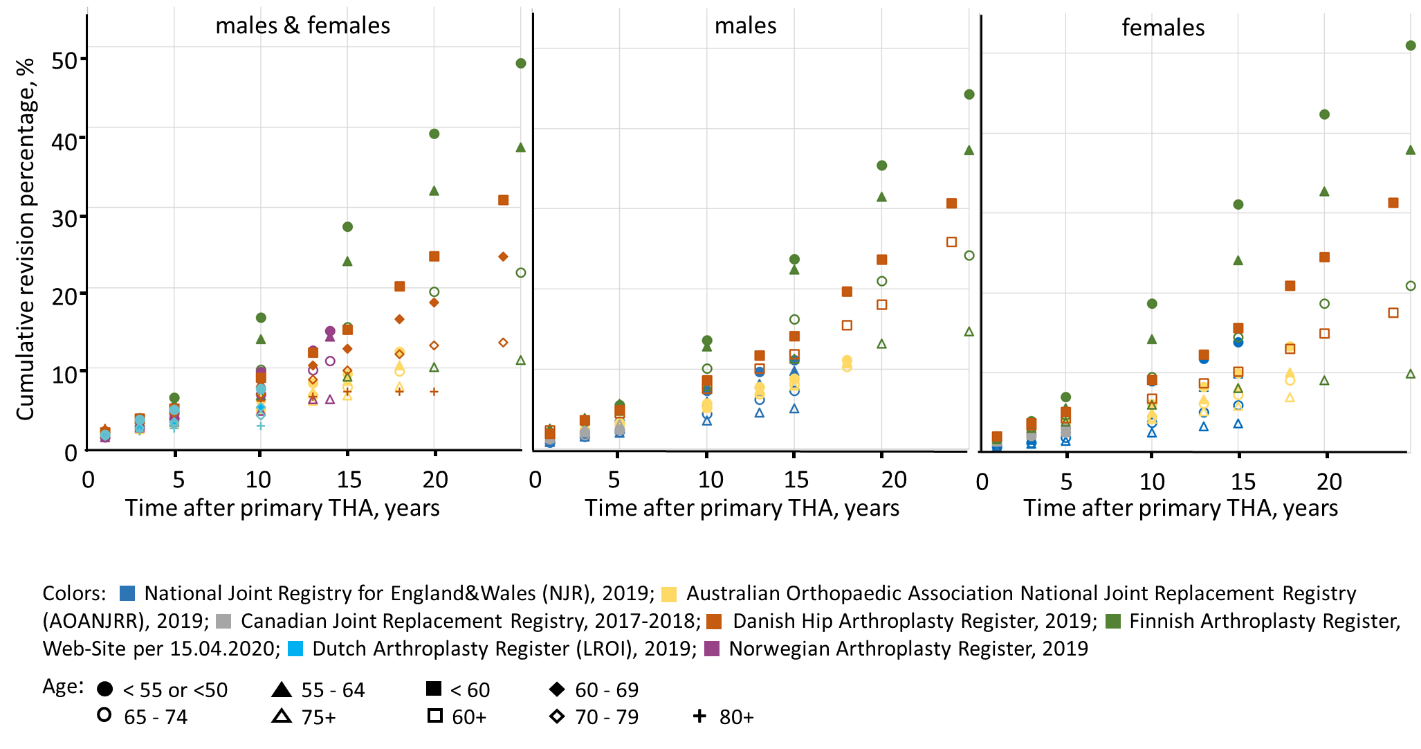
***

**Supplement 1**. Register data for cumulative revision rates in primary total hip replacement by sex and age, showing increasing revisions in younger patients compared to older patients across the registry database.
